# Chi-Nan Hu—Pioneer of Chinese neuroscience and cognitive science

**DOI:** 10.1007/s13238-019-00658-w

**Published:** 2019-10-08

**Authors:** Yunjin Wang, Yanyan Qian

**Affiliations:** 1 Center for Mental Health Education and Counseling, Taizhou University, Taizhou, 318000 China; 2 Social and Behavioral Sciences Facility, Leiden University, 2333 AK Leiden, Netherlands

**Figure 1:**
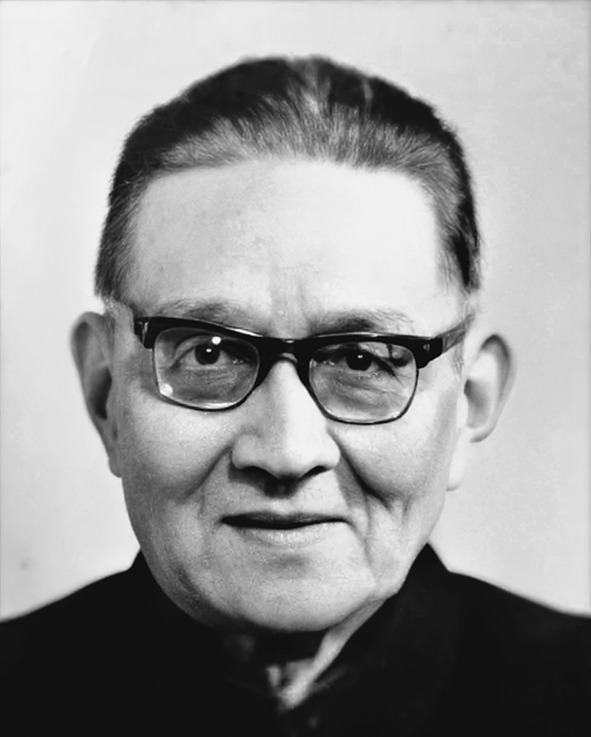
Chi-Nan Hu (1905–1989)

Chi-Nan Hu (胡寄南, 1905–1989) (Fig. 1), the renowned Chinese animal psychologist and neuropsychologist, was born in Shanghai at November 18th, 1905. He was awarded the lifetime honorary membership by the International Human Relations Training Institute (IHRT) in 1987 and became one of the first doctoral tutors in mainland China in 1988.

Hu’s research in neuroscience can be divided into two stages.

The first stage (1922–1946), Hu expanded and popularized the experimental research conducted by animal psychologists Zing-Yang Kuo (郭任远, 1898–1970) and Karl Spencer Lashley, both of whom were Hu’s supervisors at different period.

In the spring of 1922, Hu studied psychology in Fudan University supervised by Dr. Zing-Yang Kuo, a world-famous comparative psychologist and animal psychologist known as Chinese Watson, one of the most extreme precursors of the behaviorism school and the most radical behaviorist in the history of behavioristic psychology (Qian et al., 2018). Hu participated in an experimental research “genesis of the cat’s response toward the rat” and received the systematical training in the field of animal psychology research. Meanwhile, he inclined to the opposite point of the instinct theory and advocated holistic psychology. In 1925, Hu received his bachelor’s degree. It was worth noted that he was one of the existing four psychology graduates in China at that time (Hu, 1985). From then on, he devoted himself to his scientific career. Together with Sung-Kao Wu (吴颂皋) and Wei-Jung Huang (黄维荣), he translated a series of books written by Zing-Yang Kuo and published the books by the Kaiming Press in 1928, such as *“How are our instincts acquired?” “The net result of the anti-heredity movement in psychology”*.

**Figure 2:**
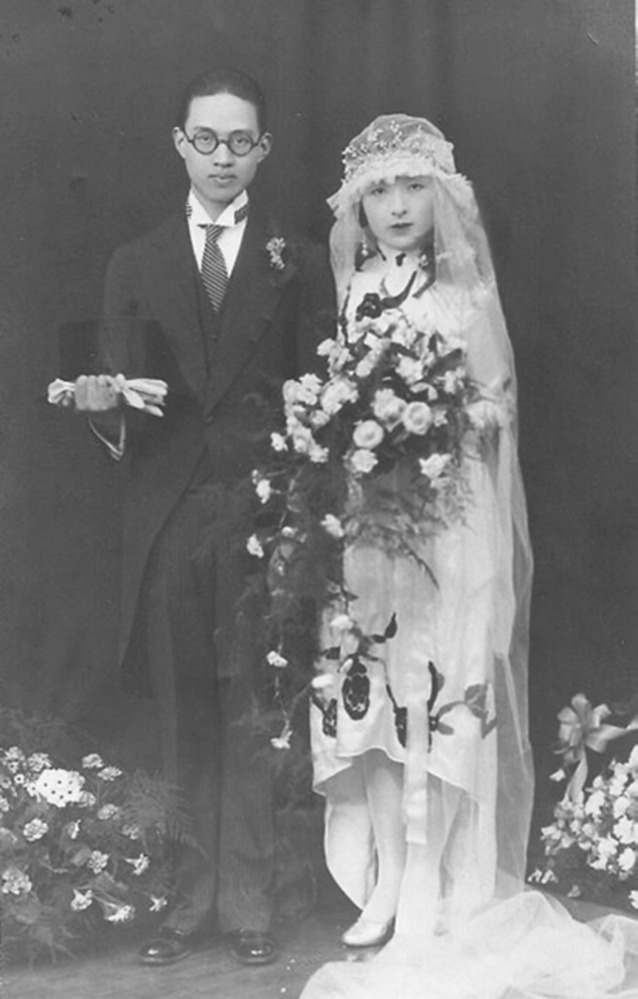
In the spring of 1930, Chi-Nan Hu married with Chu-Chen Yu (俞竹贞), a child psychologist, in Shanghai. After wedding, they both studied in the USA (http://blog.sina.com.cn/s/blog_687641d50100mv83.html#post)

**Figure 3:**
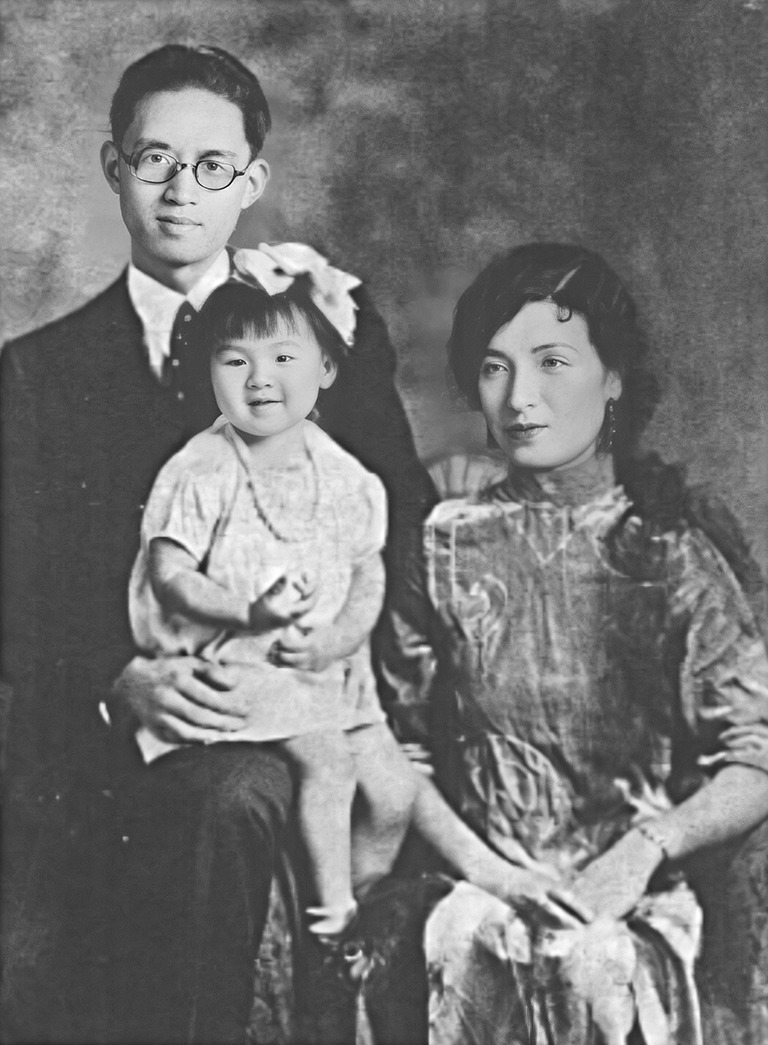
In 1932, Chi-Nan Hu, Chu-Chen Yu and their daughter (Lao S (1998) A Female general born on the other side of the ocean: Feipei Hu. Qingdao: Qingdao Publishing House)

Hu studied at Ohio State University in 1928, applied for the master’s degree in psychology in 1930 and graduated the next year (Hu, 1985; Zhou, 2015). In 1934, Hu transferred to the University of Chicago, researching in the field of memory function of the brain supervised by Karl Spencer Lashley, a famous physiological psychologist. In 1934, Hu finished his doctoral thesis on *“The effects of brain injury upon retentiveness in the rat”* (No. 18, 1938). The thesis was published in *The Journal of General Psychology* and selected as an excellent graduation thesis by the Library of Congress. This research, which pointed out cerebral lesions’s great influence on the animal memory, “established the preponderant effectiveness of the factor of cerebral lesions in contributing to the function of retention or forgetting over those of time and interpolated activity. It was in favor of the mass action of the cerebral cortex in retention as proposed by Lashley (Hu,^1938^)” (Figs. 2 and 3).

In 1936, Hu published *the brain equipotentiality and the experiments of professor Lashley* (Vol. 25, No. 4) in *Education Journal* to popularize the brain research of Lashley. Hu postulated “the experimental research of Lashley could concerning the essence of the most important issues in psychology (Hu, 1985)”. That year Hu also focused on the research methods of animal psychology, summarized and delivered a handbook, named *The methods of animal psychology research*, in which he elaborated on the goals, methods and important matters. Hu’s main goals were firstly, to satisfy curiosity; Secondly, to compare with the human psychology; thirdly, to improve practicality. Hu described three types of methods including anecdotal, observational and experimental research. Additional, he suggested there are five issues that require people to follow: firstly, raise of the problem; secondary, colloquy of history; thirdly, method of solving; fourthly, progress of the experiment (the arrangement of the instruments and the choice of materials); and finally, the results and conclusions. His thesis was included in “Reading Guide Book 1” published by Commercial Press, which is the oldest Press in China and jointly known as the “Gemini of China’s Modern Culture” with Peking University.

In 1938, Hu became a professor at Zhejiang University, and joined Jinan University as dean in 1945 (Zhou, 2015). The next year, Hu worked at China Institute of Physiology and Psychology, where he started to investigate the relationship between language and brain (Yan, 2015; General situation of China Institute of Physiology and Psychology, 1946). In 1948, he worked as a professor and dean of biology department of Fudan University. In 1951, he served as a researcher at National Institute of Education Sciences. Since 1952, he occupied the position of professor at East China Normal University (Zhou, 2015).

Since 1960s, Hu launched the second stage of his career which consists two main parts. On one hand, he translated manifold foreign literature in the field of brain research. In 1965, Hu translated *“K.S. Lashley and the integration of cerebral cortex”* written by E.G. Boring and published the book in the *Psychological Science Newsletter* which was the predecessor of *Psychological Science*. In 1981, he translated *“The Biology Foundation of Learning”*, selected from *Biopsychology*, written by James W. Kalat. The book was published on *Nature Journal* (Vol. 6, No. 8). On the other hand, he devoted himself to spreading the knowledge of physiology. After 1949, he successively taught at Fudan University and East China Normal University, with a break between 1958 and 1961 when he served as a researcher in Beijing Central Institute of Educational Science and professor of psychology at Beijing Normal University. What’s more, Hu was the member of “Five Valiant Tigers”, together with Hsiu-Rong Hsiao (萧孝嵘), Yao-Hsiang Chang (张耀翔), Jen-Hsia Tso (左任侠), Hsunchu Hsieh (谢循初), those who were psychological professors in the department of education at East China Normal University. From 1952 to 1957, Hu persisted in giving lectures on human anatomy and physiology to students majoring in education (Chen, 2018).

Hu switched research focus from behavioral psychology to cognitive psychology ever since 1960s, considering the interaction of human neurology and psychology. Hu suggests that the psychology is science about human, based on the social science and physical science. The motivating force of advancement of human psychology is the unity of contradiction between human sociology and biology (Hu, 1985). The essence of psychological activity is analysis, synthesis and storage of information (Hu, 1980). However, “the essence of information is the conversion product from physical process to neural process and then to psychological process, *vice versa*. (Hu, 1982; Hu, 1985)”. Hu mainly focused on translating and editing articles in the field of psychoanalysis in order to achieve a deep understanding of psychological activity (Wallman, 1961a, b; Hall et. al, 1961; Orser, 1963). Based on the insightful and thoughtful view of human’s neurology and psychology, he conducted the research about depressive disorder from a cognitive perspective. From 1993 to 1995, a series of articles on the subject of depression were published in collaboration with Chi-Nan Hu, Wei-Lian Zheng (郑维廉), Zhi-Liang Yang (杨治良) and Neng Cai (蔡能). These studies revealed the relationship among depression and self-respect (Zheng et. al, 1993), basic decision-making and recognition function (Zheng et. al, 1994a), imagination (Zheng et. al, 1994b), self-blame, imagination and prudence (Zheng et. al, 1995a), gender difference (Zheng et. al, 1995b).

**Figure 4:**
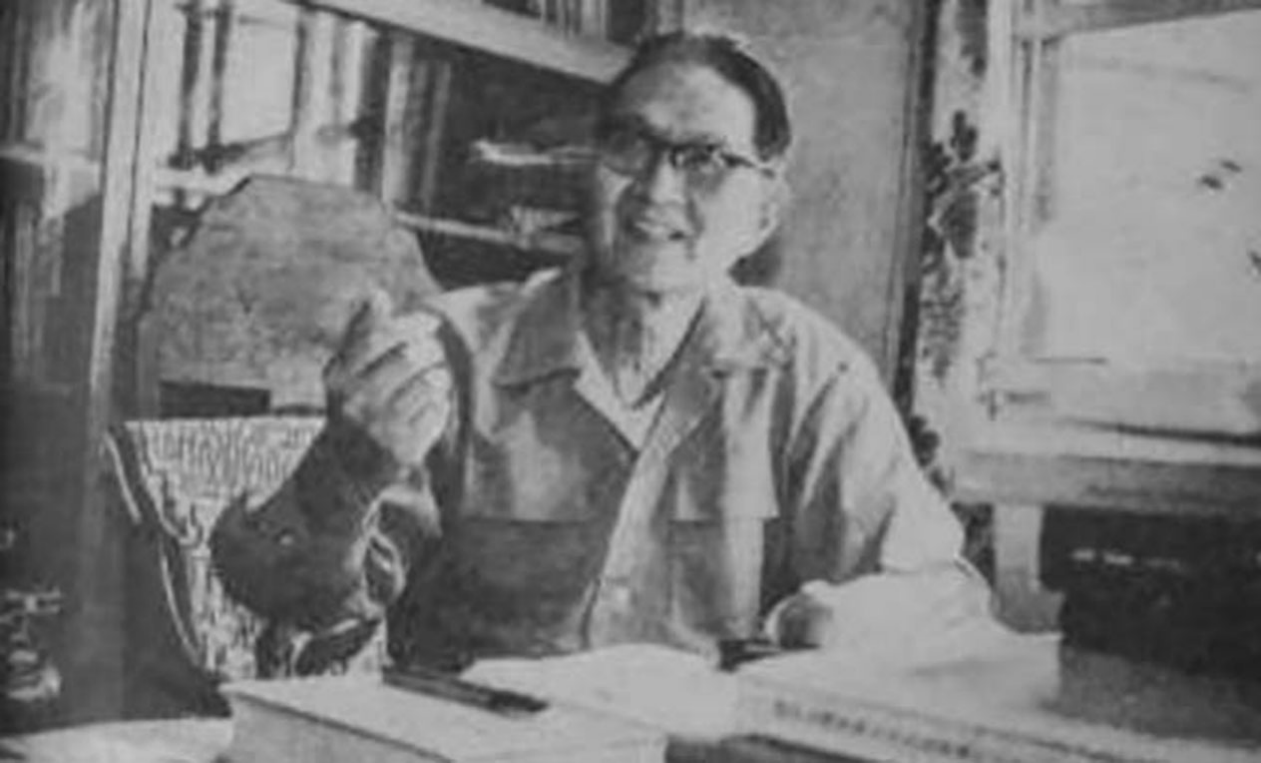
Chi-Nan Hu was doing research in his office (Hu JN (1985) A collection of selected essays on psychology: supplement edition. Shanghai: Xuelin Publishing House)

**Figure 5:**
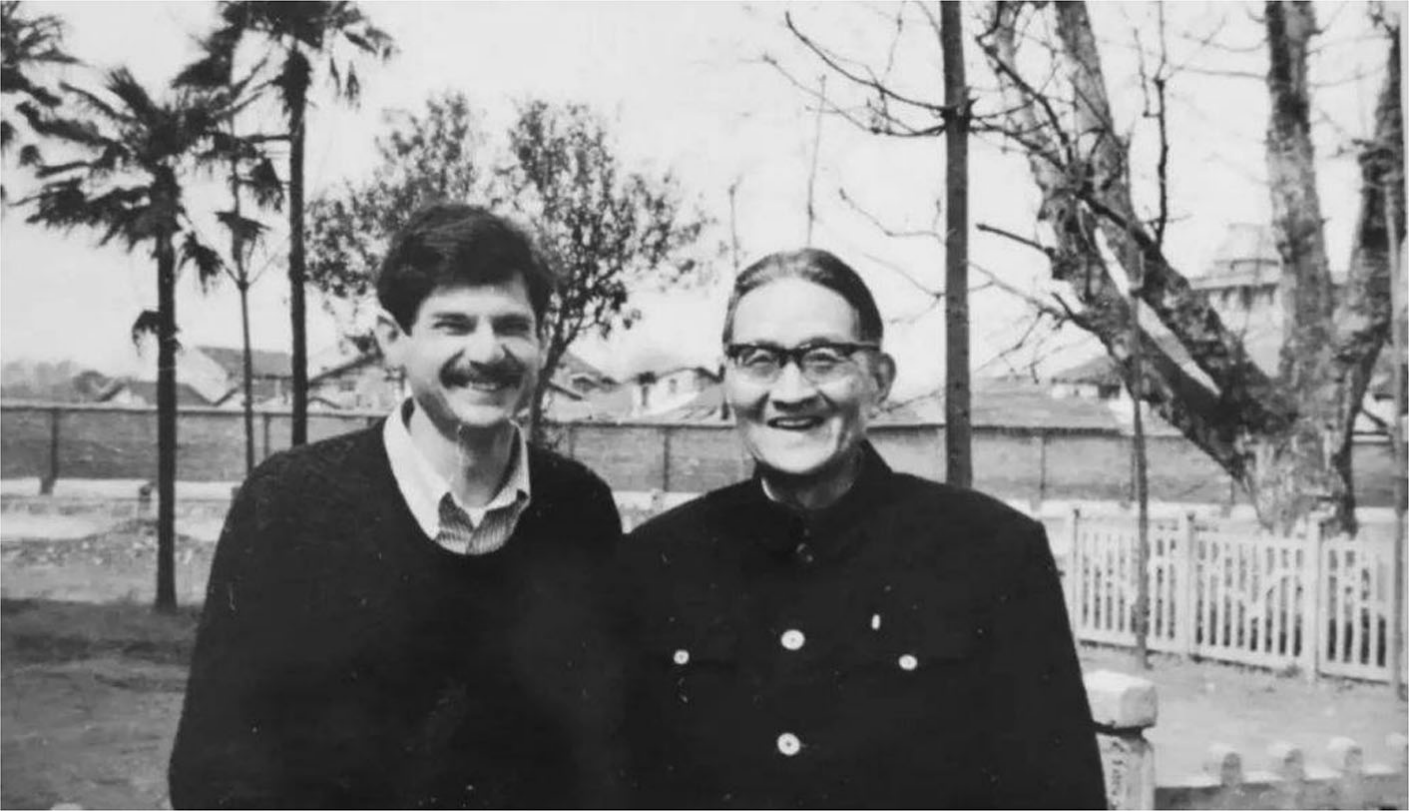
Chi-Nan Hu and his colleague, Carl Ratner (Copyright of this photo is owned by Professor Bo Wang of Nanjing University)

In 1985, at an age of 80, Hu visited the animal psychology laboratory in Boston University and met John Michael Harrison and Burrhus Frederic Skinner (Lao, 1998). Unfortunately, Hu passed away on 20th December, 1989 due to disease, at the age of 84. On the bright side he admirably lived the age of 84, he still insisted on conducting researches, translating literature and supervising his students (Figs. 4 and 5). On the evening of 6th January, 1990, a memorial meeting was held, professor Yuanlei Su (苏渊雷) wrote an elegiac couplet for Hu:

Fame and gain are lighter than feather last for all his life (人生如寄名利轻于鸿毛).

Rectitude and sincerity throughout his lifetime without any regrets (两袖清风生时遗憾少).

Hu broaden career in South which is weightier than Mount Tai (吾道其南事业重于泰山).

Open and aboveboard, influence of education will exists forever (光明磊落身后教诲长).
